# Long-Term Outcomes of Low-Dose-Rate Brachytherapy in Localized Prostate Cancer: A 17-Year Retrospective Analysis of a Single-Center Portuguese Cohort

**DOI:** 10.3390/jcm15072778

**Published:** 2026-04-07

**Authors:** Carlos Rabaça, Domingos Roda, Guy Vieira, Bruno Pereira, Ricardo Godinho, Mário Lourenço, José Alberto Pereira, Margarida Regencio, Sofia Macedo, Amilcar Sismeiro

**Affiliations:** 1 Center for the Treatment of Urological Diseases, R. Dom Manuel I n° 8, Estádio Cidade de Coimbra, 3030-320 Coimbra, Portugal; domingosmroda@gmail.com (D.R.); guy.p.vieira@gmail.com (G.V.); 4076@ipocoimbra.min-saude.pt (B.P.); 2243@ipocoimbra.min-saude.pt (R.G.); 3685@ipocoimbra.min-saude.pt (M.L.); 4023@ipocoimbra.min-saude.pt (J.A.P.); 3345@ipocoimbra.min-saude.pt (M.R.); scapelamacedo@gmail.com (S.M.); geral@cetruc.pt (A.S.); 2 Portuguese Institute of Oncology of Coimbra, Av. Bissaya Barreto 98, 3000-075 Coimbra, Portugal; 3 Faculty of Medicine, University of Coimbra, Azinhaga de Santa Comba, Celas, 3000-548 Coimbra, Portugal; 4 Joaquim Chaves Saúde, Rua Aníbal Bettencourt, n° 3, Edifício CORE, 2790-225 Carnaxide, Portugal; 5 Faculty of Health Sciences, University Beira Interior, Av. Infante D. Henrique, 6200-506 Covilhã, Portugal; 6 Hospital Lusíadas, Av. dos Hospitais Civis de Lisboa 8, 2724-002 Amadora, Portugal

**Keywords:** prostate cancer, brachytherapy, overall survival, biochemical recurrence-free survival, complications

## Abstract

**Background/Objectives**: Prostate cancer is one of the most common malignancies diagnosed in men worldwide. Brachytherapy (BT), particularly low-dose rate (LDR)-BT, has been shown to be a successful treatment. The aim of this study was to evaluate the effectiveness of BT treatment in localized prostate cancer patients from a single-center Portuguese cohort. **Methods**: This was a retrospective study that evaluated prostate cancer patients followed up at the Center for the Treatment of Urological Diseases, Coimbra, Portugal, who underwent LDR-BT between November 2007 and March 2024. Overall survival (OS), biochemical recurrence-free survival (BRFS) and complications post-LDR BT treatment were evaluated during patients’ follow-up time. **Results**: A total of 1343 patients treated with LDR-BT were recruited. Global OS and BRFS rates were 98.4% and 87.7%, respectively. A reduced frequency of complications such as lower urinary tract symptoms, erectile dysfunction, acute urinary retention, radiation proctitis and stress urinary incontinence were described. High OS (>98%) and BRFS rates were observed particularly in low and intermediate disease risk. Prostate-specific antigen (PSA) serum levels > 20 ng/mL, Gleason score (GS) ≥ 8 and clinical tumor stage (cT) ≥ T2c were identified as the strongest predictors of death and/or biochemical recurrence. **Conclusions**: BT is an effective treatment in localized prostate cancer patients, with comparable outcomes and consistent with the OS and BRFS rates reported in the current literature for radical prostatectomy and external beam radiotherapy approaches, and with a reduced frequency of complications. PSA serum levels > 20 ng/mL, GS ≥ 8 and cT stage ≥ T2c can be used as strong predictors of death and/or biochemical recurrence during patients’ follow-up.

## 1. Introduction

Prostate cancer is the second-most common malignancy diagnosed in men, and the second leading cause of cancer death in men after lung cancer [1,2,3].

Prostate cancer risk increases strongly with age [2,3,4]. Other risk factors associated with prostate cancer development and/or higher mortality rates include ethnicity [5], obesity [6], a predominantly Western diet [7] and exposure to cigarette smoke [8]. Furthermore, genetic factors have also been linked to an increased risk of early-onset disease [9].

Treatment of prostate cancer varies based on how advanced the cancer is, the risk of metastasis, the patient’s health, and preferences. Notably, treatment decisions depend on the risk of biochemical recurrence (BCR), which is estimated according to the D’Amico risk stratification that classifies patients into low-, intermediate-, and high-risk groups based on prostate-specific antigen (PSA) levels, clinical tumor stage (cT), and Gleason score (GS) at diagnosis [10]. Depending on patient risk classification, treatment options can include active surveillance/or watchful waiting, surgery (radical prostatectomy), radiotherapy (external beam radiotherapy—EBRT, or brachytherapy—BT), androgen deprivation therapy, second-generation androgen receptor-targeting agents, or chemotherapy [2,4].

BT, using low-dose-rate (LDR) permanent seed implantation or high-dose-rate (HDR) temporary source implantation, is an effective treatment option for selected patients with prostate cancer of any risk group [11,12]. Particularly, LDR-BT has been shown to be a successful treatment in patients with localized prostate cancer, with reduced side effects and being less invasive when compared to surgery or EBRT, improving patients’ quality of life [13,14].

In Portugal, the number of BT procedures has significantly increased in the last 25 years [15], but the knowledge regarding the effects of this treatment is still scarce. Therefore, the aim of this study was to evaluate the effectiveness of LDR-BT in localized prostate cancer patients when compared to published data in the literature about radical prostatectomy (open, laparoscopic, or robotic-assisted) and EBRT. For that, overall survival (OS), biochemical recurrence-free survival (BRFS) and complications post-LDR-BT treatment were evaluated during patients’ follow-up time.

## 2. Materials and Methods

### 2.1. Patient Recruitment

This was a retrospective study that evaluated prostate cancer patients followed up at the Center for the Treatment of Urological Diseases, Coimbra, Portugal, who underwent LDR-BT between 13 November 2007 and 18 March 2024. For clinical characterization of patients, a group of variables was collected, including age, PSA serum levels, GS, cT, time until BCR, and time until death. Patients were classified according to D’Amico risk stratification, and clinical data were collected based on D’Amico risk stratification parameters (PSA serum levels, GS, and cT). OS, BRFS, and complications described by patients after LDR-BT treatment were registered during follow-up. Patients who had been previously treated with surgery (radical prostatectomy) or EBRT were excluded.

### 2.2. Ethical Considerations

This study was performed in accordance with the ethical standards of the Declaration of Helsinki, and its subsequent amendments, and it was approved by the Ethics Committee of Faculty of Medicine, Coimbra, Portugal.

### 2.3. Statistical Analysis

Survival curves were calculated by the Kaplan–Meier method and compared using the Log-Rank test. A Cox regression analysis was used for comparison between groups. All statistical analysis of data was performed using IBM SPSS Statistics 31.0.1.0 (IBM Corporation, Armonk, NY, USA). Statistical significance was set at *p* < 0.05.

## 3. Results

### 3.1. Clinical Characterization of Patients

A total of 1343 prostate cancer patients who underwent LDR-BT were included. Patients had a median age of 68 years (range 40–91) and the median OS time was 16.7 years. Patients were classified according to D’Amico risk stratification as low (N = 531, 41.8%), intermediate (N = 632, 49.8%) and high (N = 107, 8.4%) disease risk. Initial PSA serum levels had a median value of 6.64 ng/mL (range 0.19–173.0) and, after D’Amico risk stratification, a total of 1024 patients (79.9%) had PSA serum levels < 10 ng/mL; 198 patients (15.5%) had PSA serum levels between 10 and 20 ng/mL; 59 patients (4.6%) had PSA serum levels ≥ 20 ng/mL. The median GS was seven (range five to ten) and, after D’Amico risk stratification, a total of 641 patients (49.8%) had GS ≤ 6; 602 patients (46.7%) had GS = 7; and 45 patients (3.5%) had GS ≥ 8. Patients were also classified according to cT based on D’Amico risk stratification as cT1–T2a (N = 1131, 88.1%); cT = T2b (N = 97, 7.6%) and cT ≥ 2c (N = 56, 4.4%). The median time that occurred either until BCR or death was 8.0 years (range 0–17). Clinical characterization of all patients included in this study is described in Table 1.

### 3.2. Overall Survival of Patients After LDR-BT

**Figure 1:**
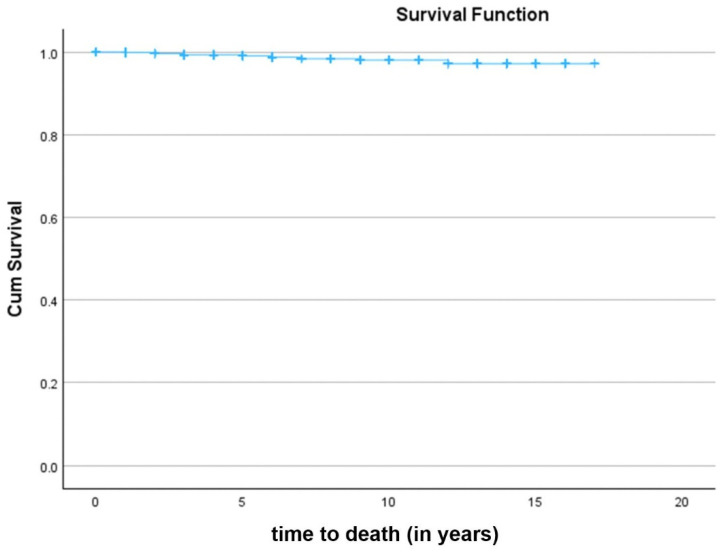
Kaplan–Meier curve of overall survival (OS) of prostate cancer patients after low-dose rate brachytherapy (LDR-BT). Differences were considered statistically significant for *p* < 0.05.

**Figure 2:**
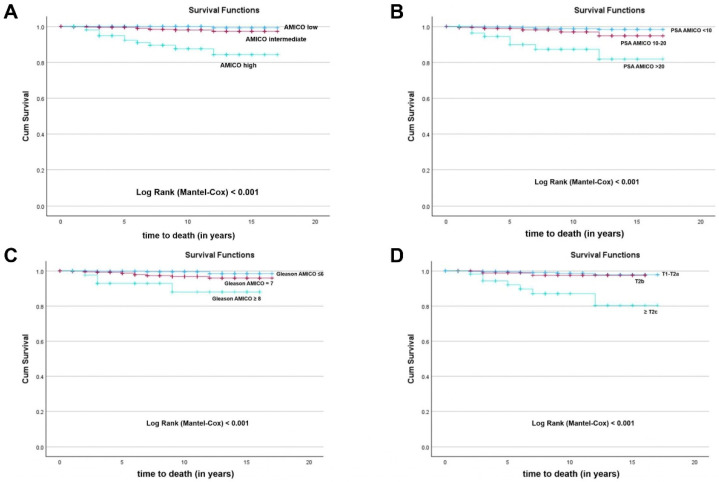
Kaplan–Meier curves of overall survival (OS) of prostate cancer patients after low-dose rate brachytherapy (LDR-BT) classified according to the D’Amico risk stratification (**A**); prostate-specific antigen (PSA) serum levels based on D’Amico risk stratification (**B**); Gleason score (GS) based on D’Amico risk stratification (**C**); clinical tumor stage (cT) based on D’Amico risk stratification (**D**). Differences were considered statistically significant for *p* < 0.05.

The OS of patients after LDR-BT was 98.4% (Figure 1). Statistically significant differences were observed in OS analysis of patients after D’Amico risk stratification (Log-Rank test, *p* < 0.001) (Figure 2A). Low-risk patients had an OS of 99.8% when compared to intermediate-risk (98.6%) and high-risk patients (89.7%). Cox regression analysis showed that high and intermediate-risk patients had a higher risk of death (hazard ratio (HR) 55.75; 95% confidence interval (CI): 7.197–431.899; *p* < 0.001 and HR 8.26; 95% CI: 1.046–65.211; *p* = 0.045, respectively) when compared with low-risk patients. Statistically significant differences were also found in OS results when analyzing patients according to D’Amico risk stratification parameters, namely PSA serum levels (Figure 2B), GS (Figure 2C), and cT stage (Figure 2D). Patients with PSA values < 10 ng/mL had an OS of 99.1% when compared to patients with PSA levels between 10 and 20 ng/mL (97.5%) and patients with PSA levels > 20 ng/mL (88.1%) (Log-Rank test, *p* < 0.001) (Figure 2B). Cox regression analysis showed that patients with PSA levels > 20 ng/mL (HR 12.95; 95% CI: 4.823–34.787; *p* < 0.001) had a higher risk of death when compared with patients with PSA values < 10 ng/mL. Nevertheless, no significant differences were found between patients with PSA levels between 10 and 20 ng/mL (HR 2.84; 95% CI: 0.950–8.459; *p* = 0.062) and patients with PSA values < 10 ng/mL. Moreover, patients with GS ≤ 6 had an OS of 99.4% when compared to patients with GS = 7 (97.8%) and GS ≥ 8 (91.1%) (Log-Rank test, *p* < 0.001) (Figure 2C). Cox regression analysis showed that patients with GS ≥ 8 (HR 14.60; 95% CI: 3.650–58.415; *p* < 0.001) and GS = 7 (HR 4.13; 95% CI: 1.343–12.674; *p* = 0.013) had a higher risk of death when compared to patients with GS ≤ 6. Furthermore, patients with cT1–T2a had an OS of 98.9% when compared to patients with cT = T2b (97.9%) and cT ≥ T2c (87.5%) (Log-Rank test, *p* < 0.001) (Figure 2D). Cox regression analysis showed that patients with cT ≥ T2c (HR 10.95; 95% CI: 4.309–27.806; *p* < 0.001) had a higher risk of death when compared with patients with cT1–T2a. Nonetheless, no significant differences were found between patients with cT = T2b (HR 1.47; 95% CI: 0.328–6.599; *p* = 0.614) and patients with cT1–T2a.

In addition, patients with PSA serum levels > 20 ng/mL (HR 5.55; 95% CI: 1.830–16.839; *p* = 0.002), GS ≥ 8 (HR 4.58; 95% CI: 1.013–20.754; *p* = 0.048), and cT stage ≥ T2c (HR 4.61; 95% CI: 1.581–13.448; *p* = 0.005) were identified as the strongest predictors of death with similar prediction.

### 3.3. Biochemical Recurrence-Free Survival of Patients After LDR-BT

BCR was defined in accordance with the Phoenix criteria, which considers that BCR occurs after radiotherapy when a PSA rise of at least 2 ng/mL above nadir occurs [16].

**Figure 3:**
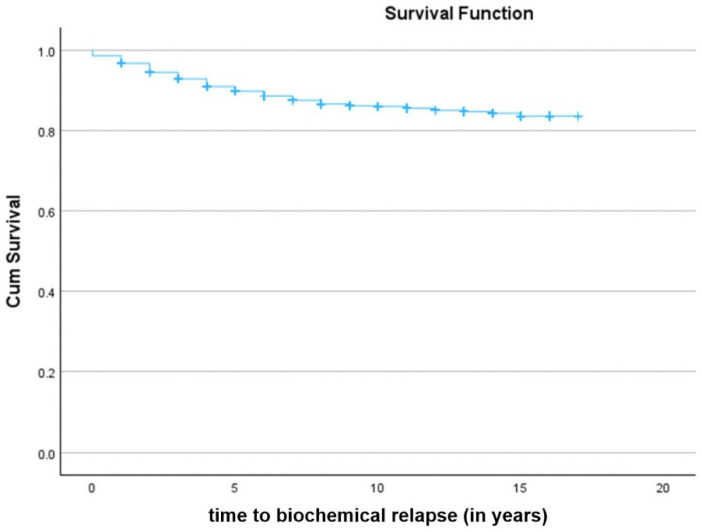
Kaplan–Meier curve of global biochemical recurrence-free survival (BRFS) of prostate cancer patients after low-dose rate brachytherapy (LDR-BT). Differences were considered statistically significant for *p* < 0.05.

**Figure 4:**
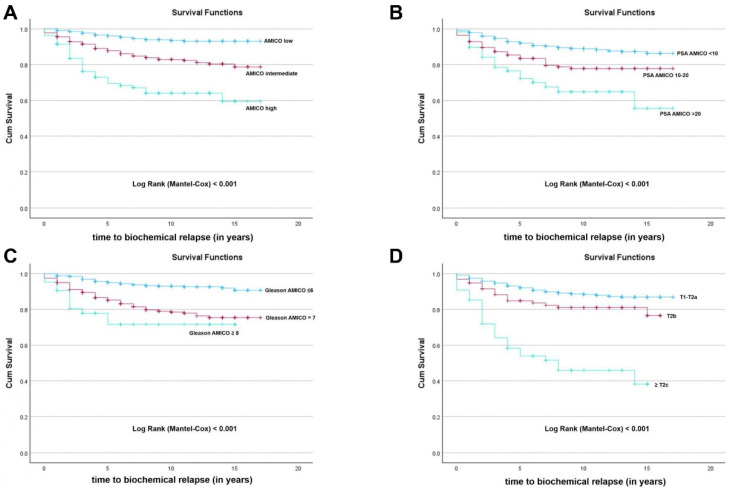
Kaplan–Meier curve of biochemical recurrence-free survival (BRFS) of prostate cancer patients after low-dose rate brachytherapy (LDR-BT) classified according to the D’Amico risk stratification (**A**); prostate-specific antigen (PSA) serum levels based on D’Amico risk stratification (**B**); Gleason score (GS) based on D’Amico risk stratification (**C**); clinical tumor stage (cT) based on D’Amico risk stratification (**D**). Differences were considered statistically significant for *p* < 0.05.

Regarding BRFS of patients after LDR-BT, a total of 1270 patients were analyzed. The median BRFS time was 15.0 years. The global BRFS after LDR-BT was 87.7% (Figure 3). Statistically significant differences were detected in BRFS of patients after D’Amico risk stratification (Log-Rank test, *p* < 0.001) (Figure 4A). Low-risk patients had a BRFS of 94.5% when compared to intermediate-risk (85.3%) and high-risk patients (66.7%). Cox regression analysis showed that high and intermediate-risk patients had a higher risk of BCR (HR 7.07; 95% CI: 4.319–11.564; *p* < 0.001, and HR 3.04; 95% CI: 2.004–4.622; *p* < 0.001, respectively) when compared with low-risk patients. Also, statistically significant differences were found in BRFS when analyzing patients according to D’Amico risk stratification parameters, namely PSA serum levels (Figure 4B), GS (Figure 4C) and cT stage (Figure 4D). Patients with PSA values < 10 ng/mL had a BRFS of 90.2% when compared to patients with PSA levels between 10 and 20 ng/mL (80.9%) and patients with PSA levels > 20 ng/mL (67.2%) (Log-Rank test, *p* < 0.001) (Figure 4B). Cox regression analysis showed that patients with PSA levels > 20 ng/mL (HR 3.69; 95% CI: 2.255–6.022; *p* < 0.001) and PSA levels between 10 and 20 ng/mL (HR 2.04; 95% CI: 1.400–2.980; *p* < 0.001) had a higher risk of BCR when compared with patients with PSA values < 10 ng/mL. Moreover, patients with GS ≤ 6 had a BRFS of 93.5% when compared to patients with GS = 7 (82.4%) and GS ≥ 8 (73.8%) (Log-Rank test, *p* < 0.001) (Figure 4C). Cox regression analysis showed that patients with GS ≥ 8 (HR 4.77; 95% CI: 2.452–9.289; *p* < 0.001) and GS = 7 (HR 3.25; 95% CI: 2.264–4.675; *p* < 0.001) had a higher risk of BCR when compared with patients with GS ≤ 6. Furthermore, patients with cT1–T2a had a BRFS of 90.1% when compared to patients with cT = T2b (81.1%) and cT ≥ T2c (48.1%) (Log-Rank test, *p* < 0.001) (Figure 4D). Cox regression analysis showed that patients with cT ≥ T2c and cT = T2b had a higher risk of BCR (HR 6.33; 95% CI: 4.177–9.586; *p* < 0.001 and HR 1.74; 95% CI: 1.056–2.864; *p* = 0.030, respectively) when compared with patients with cT1–T2a.

In addition, we found that the strongest predictors of BCR were by decreasing order: cT stage ≥ T2c (HR 4.35; 95% CI: 2.756–6.862; *p* < 0.001); GS = 7 (HR 2.71; 95% CI: 1.871–3.915; *p* < 0.001); GS ≥ 8 (HR 2.34; 95% CI: 1.153–4.732; *p* = 0.019); PSA serum levels > 20 ng/mL (HR 1.97; 95% CI: 1.164–3.319; *p* = 0.011); PSA serum levels between 10 and 20 ng/mL (HR 1.71; 95% CI: 1.167–2.499; *p* = 0.006).

### 3.4. A Reduced Frequency of Complications Is Described After LDR-BT

**Figure 5:**
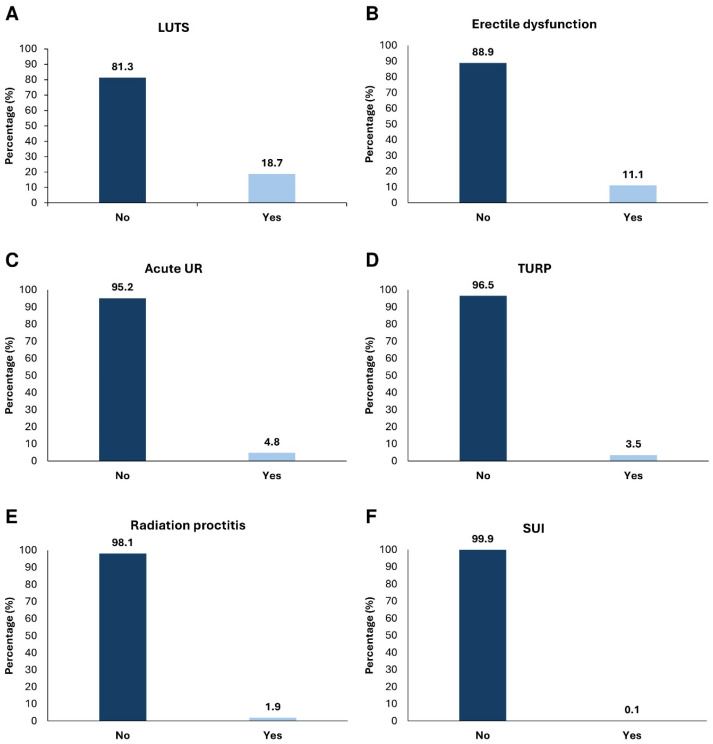
Complications described by prostate cancer patients after low-dose rate brachytherapy (LDR-BT). Percentage of patients with lower urinary tract symptoms (LUTS) (**A**); erectile dysfunction (ED) (**B**); acute urinary retention (UR) (**C**); submitted to transurethral resection of the prostate (TURP) (**D**); radiation proctitis (**E**); with stress urinary incontinence (SUI) after LDR-BT (**F**). Differences were considered statistically significant for *p* < 0.05.

Complications associated with prostate cancer development were described by a minority of patients after treatment with LDR-BT (Figure 5). Lower urinary tract symptoms (LUTS) were described by 18.7% (N = 251) of patients who did not have this symptom during initial diagnosis, but who had developed it after LDR-BT and lasted more than one year post-treatment (Figure 5A). Erectile dysfunction (ED) was considered a complication if it occurred during the first 5 years after LDR-BT, and it was described by 11.1% (N = 149) of patients (Figure 5B). Furthermore, 4.8% (N = 64) of patients mentioned acute urinary retention (UR) after LDR-BT (Figure 5C). Also, 3.5% (N = 47) of patients had to be submitted to transurethral resection of the prostate (TURP) post-LDR-BT for prostatic urethral catheter removal, or LUTS resistant to treatment (Figure 5D). Radiation proctitis post-treatment was considered in symptomatic patients (rectorrhagia), and it was described by 1.9% (N = 25) of patients (Figure 5E). Of note, two patients developed recto-urethral fistulas. Also, 0.1% (N = 2) of patients mentioned having stress urinary incontinence (SUI) after LDR-BT (Figure 5F).

## 4. Discussion

Prostate cancer is one of the most common malignancies diagnosed in men worldwide and a leading cause of cancer-related deaths in men each year. Localized disease is confined to the prostate and accounts for approximately 75% of new diagnoses [17]. The standard treatments for localized prostate cancer are RP, EBRT and BT. Active surveillance or observation is also an option in case of an indolent disease course. Nevertheless, clinical established guidelines recommend that decisions regarding treatment options should be based on a comprehensive evaluation of cancer features, including cancer staging, baseline PSA levels, patient age, comorbidity, life expectancy, and quality of life [18,19,20,21].

In Portugal, prostate cancer incidence has been rising since 1998 [22]. In 2022, prostate cancer was the most frequent malignancy diagnosed in Portuguese men, with 7529 new incident cases and 2083 deaths reported [23]. Since 2000, the number of BT treatment procedures in Portugal has significantly increased [15].

Overall, our results support that BT is an effective treatment in localized prostate cancer patients with an OS and BRFS of 98.4% and 87.7%, respectively, and with a reduced frequency of complications such as LUTS, erectile dysfunction, acute UR, radiation proctitis and SUI. Furthermore, high OS (>98%) and BRFS rates were also observed when patients were classified according to the D’Amico risk stratification, particularly in low and intermediate disease risk. Moreover, when patients were evaluated according to D’Amico risk stratification parameters, the lowest OS and BRFS rates were significantly associated with PSA serum levels > 20 ng/mL, GS ≥ 8 and cT stage ≥ T2c. In addition, PSA serum levels > 20 ng/mL, GS ≥ 8 and cT stage ≥ T2c were identified as the strongest predictors of death and/or BCR in these patients.

The efficacy of BT treatment in OS and BRFS rates presented herein is further supported by additional studies in the literature [12,24,25,26,27,28,29]. Indeed, BT remains the main treatment option for patients with localized prostate cancer, with 10-year survival data showing favorable outcomes [30,31,32]. A report by Kittel et al. [30] with long-term follow-up of LDR BT as monotherapy in prostate cancer patients has been shown that the 5-year and 10-year OS were 93.7% and 76.1%, respectively. Furthermore, it was also documented that the 5-year and 10-year BRFS were 91.9% and 81.5%, respectively. Similarly, a report on 10-year experience of permanent BT monotherapy at a single UK center has shown an OS rate of 85% [32]. In addition, long-term results of a phase II clinical trial of transrectal ultrasound-guided permanent radioactive implantation of the prostate have shown an 8-year PSA control of 92% and an OS of 88% [33], which is also consistent with the long-term results of EBRT or surgery for a similar population of organ-confined prostate cancer patients [34,35,36]. In fact, it has been shown that low and intermediate disease risk patients treated with LDR-BT have similar survival rates to those found with surgery (RP) or EBRT, and with fewer side effects such as urinary incontinence and sexual dysfunction [37,38,39,40,41,42]. Moreover, BT has also been associated with significantly lower long-term toxicities [27,30,43,44].

Results from the Prostate Testing for Cancer and Treatment (ProtecT) trial, a large, UK-based study that compared active monitoring, RP and radiotherapy for men with clinically localized prostate cancer, suggest that radiotherapy offers similar outcomes and improved toxicity and quality of life over surgery [45]. After 15 years of follow-up, the study found no significant difference in death from prostate cancer between the three groups. Nonetheless, patients on active monitoring had a higher risk of their cancer progressing or metastasizing compared to those who received RP or radiotherapy. Additionally, no differential effects on cancer-specific mortality were noted in relation to the baseline PSA level, tumor stage, or grade, or risk-stratification score. Also, a randomized controlled trial on BT and RP reported similar 5-year BRFS rates for BT (91.7%) and RP (91.0%) [46]. Notably, a report on 15-year experience on LDR-BT in prostate cancer patients in Japan has shown excellent oncological outcomes of LDR-BT, with equivalent or superior efficacy to surgery in low- to intermediate-risk patients, and superior efficacy in high-risk patients [43]. Recently, the oncological and functional outcomes of robot-assisted RP (RARP) and BT were compared in a single-center prospective randomized study and similar BRFS rates were found after BT and RARP [47].

In addition, studies comparing the oncological outcomes of BT and EBRT have also demonstrated similar OS and no significant differences in 5-year BRFS rates [48,49]. Moreover, better scores for bowel function were reported for LDR-BT when compared to EBRT [50]. In fact, a recent study by Andruska et al. has shown that the addition of BT to EBRT correlated with improved survival in men with unfavorable intermediate-risk prostate cancer [51].

LDR-BT is also frequently combined with other treatments to enhance efficacy, particularly in intermediate- and high-risk prostate cancer patients. Besides EBRT, some of the most common drug combinations with LDR-BT include ADT [Luteinizing Hormone-Releasing Hormone (LHRH) agonists/antagonists; anti-androgens], the most standard systemic treatment used alongside LDR-BT; and alpha-blockers, commonly used prophylactically to reduce the risk of urinary toxicity and LUTS following seed implantation [52,53,54,55]. Furthermore, emerging studies investigating the addition of novel androgen receptor pathway inhibitors to standard radiation therapy, particularly in high-risk prostate cancer patients, are currently ongoing with promising results [56,57,58]. Moreover, recent advances are being developed to explore the potential benefits of combining BT and immunotherapies [59,60,61,62].

Despite the progress achieved so far in therapeutic approaches for prostate cancer, treatment resistance can occur and is often influenced by the complexity of the prostate tumor microenvironment (TME). Indeed, the prostate TME is a complex dynamic network that involves the interaction of different cellular components, including stromal cells, immune cells, blood vessels, and extracellular matrix, that profoundly influence disease progression and treatment outcomes since it creates an immunosuppressive, acidic, and hypoxic niche that protects cancer cells and promotes castration resistance [63,64,65]. Therefore, understanding the interaction between prostate cancer cells and their microenvironment is crucial for developing better prognosis tools and personalized therapies [59,60,66]. In fact, a recent study by Peng et al. has identified a group of aneuploidy driver genes related to the immune microenvironment, development and metastasis of prostate cancer, which could be promising therapeutic targets [67]. Furthermore, nine docetaxel-specific chemoresistant genes related to immune infiltration in prostate cancer have been recently identified that could serve as potential biomarkers or drug targets for the combination of docetaxel [68]. Also, a recent study by Li et al. based on multi-omics data has established a prognostic model that not only stratifies patients into high- and low-risk groups but also highlights subtype-specific drug resistance patterns [63]. Of note, it has been recently suggested that combining BT with immune checkpoint inhibitors holds significant promise for enhancing cancer treatment through precise high-dose targeting and immune system activation [59].

Although the European Association of Urology (EAU) guidelines recommend active surveillance for patients with low-risk prostate cancer and a life expectancy greater than 10 years, we still have some reservations regarding this strategy. First, even low-risk diseases may occasionally show an unexpectedly aggressive biological behavior. Second, in our clinical experience, active surveillance generates significant anxiety in many patients, particularly in Mediterranean/Latin populations, where the cultural acceptance of living with an untreated cancer is often limited. Finally, when an effective treatment is available that is minimally invasive and associated with very low morbidity (such as BT), it is reasonable to consider active treatment. This is particularly relevant in the context of increasing life expectancy, where long-term oncological control becomes even more important. Therefore, based on our clinical experience, we recommend BT treatment also for patients with low-risk prostate cancer and a life expectancy greater than 10 years.

Some limitations of our study need to be considered in data interpretation, such as the lack of objective Patient-Reported Outcome Measures (PROMs)/Patient-Reported Experience Measures (PREMs) for functional outcomes. Since this is a retrospective study with data collection initiated in 2007, standardized and validated questionnaires (PROMs and PREMs such as IPSS or IIEF) were not systematically utilized or recorded across all patients during the earlier years of the cohort. Consequently, complications such as LUTS and erectile dysfunction were assessed based on clinical reporting documented in the patients’ medical records rather than objective scoring tools. Nonetheless, we considered the occurrence of LUTS in patients who had no previous symptoms and developed them after the procedure, requiring alpha-blocker medication for more than one year. Furthermore, regarding erectile dysfunction, we considered it to be a side effect in patients who reported it within the five years following the procedure and required medication. Moreover, patient follow-up was always carried out by the same group of urologists, which ensured a consistent and homogeneous assessment.

In addition, some of the patients included in our cohort had large prostate volumes, which fall outside standard international guidelines for LDR-BT that typically recommend smaller volumes to avoid pubic arch interference and severe urinary toxicity. Nevertheless, in our cohort, a minimal number of patients with highly enlarged prostates were treated as exceptions, with no significant pubic arch interference.

To the best of our knowledge, this is the first study in Portugal that has analyzed the effects of BT treatment in a large cohort of prostate cancer patients based on a single-center analysis. Future studies with a multicenter national approach and collaborations are required.

## 5. Conclusions

To sum up, our results suggest that BT is an effective treatment in prostate cancer patients, with comparable outcomes and consistent with the OS and BRFS rates reported in the current literature for RP and EBRT approaches, and with a reduced frequency of complications. Furthermore, high OS and BRFS rates after BT treatment were particularly observed in low and intermediate-risk patients. In addition, PSA serum levels > 20 ng/mL, GS ≥ 8, and cT stage ≥ T2c were identified as the strongest predictors of death and/or BCR.

## Figures and Tables

**Table 1:** Clinical characterization of prostate cancer patients.

Variable	Median	Range (Min–Max)
Age (years)	68	40–91
Overall survival time (years)	16.7	0–17
Time until biochemical recurrence (years)	8.0	0–17
Time until death (years)	8.0	0–17
Prostate volume (cc)	40.0	11.0–143.0
**D’ Amico classification (N, %)**		
Low risk	531 (41.8%)	
Intermediate risk	632 (49.8%)	
High risk	107 (8.4%)	
**PSA serum levels (ng/mL)**	6.64	0.19–173.0
PSA < 10 (n, %)	1024 (79.9%)	
PSA 10–20 (n, %)	198 (15.5%)	
PSA > 20 (n, %)	59 (4.6%)	
**Gleason score (GS)**	7	5–10
GS ≤ 6 (n, %)	641 (49.8%)	
GS = 7 (n, %)	602 (46.7%)	
GS ≥ 8 (n, %)	45 (3.5%)	
**Clinical tumor stage (cT) (N, %)**		
cT1–T2a	1131 (88.1%)	
cT = T2b	97 (7.6%)	
cT ≥ 2c	56 (4.4%)	

## Data Availability

The data presented in this study are available from the corresponding author on reasonable request.
